# A streamlined predictive model for predicting the risk of recurrence after liver transplantation for hepatocellular carcinoma was constructed based on preoperative ^18^F-FDG PET/CT metabolic parameters and clinicopathological features

**DOI:** 10.3389/fonc.2026.1832413

**Published:** 2026-07-27

**Authors:** Xianglei Kong, Sibo Zhou, Shengli Ye, Zhengye Cao, Jiaqi Wang

**Affiliations:** 1 Department of Radiology, Sir Run Run Shaw Hospital, Zhejiang University School of Medicine, Hangzhou, China; 2 Department of Radiology, The First People’s Hospital of Yuhang District, Hangzhou, China; 3 Department of Nuclear Medicine and Radiology, Shulan (Hangzhou) Hospital, Shulan International Medical College, Zhejiang Shuren University, Hangzhou, China

**Keywords:** ^18^F-FDG PET/CT, clinicopathological features, hepatocellular carcinoma, liver transplantation, metabolic parameters, predictive model, risk of recurrence

## Abstract

**Objective:**

This study aimed to combine preoperative fluorodeoxyglucose (^18^F-FDG) positron emission tomography/computed tomography (PET/CT) metabolic parameters with postoperatively available clinicopathological features to construct a streamlined predictive model for recurrence risk after liver transplantation for hepatocellular carcinoma (HCC), providing a basis for individualized recurrence risk assessment and guiding diagnosis and treatment strategies.

**Methods:**

This retrospective study included 176 HCC transplant recipients with preoperative ^18^F-FDG PET/CT, with a median follow-up of 12 months (range: 6–61 months). Clinicopathological and PET/CT metabolic data were collected. Univariate Cox regression screened for recurrence-related factors. After collinearity diagnosis (VIF > 10), PET parameters (coefficient of variation [COV] and total lesion glycolysis [TLG]) were manually selected and combined with Boruta-screened clinicopathological features to construct a multivariate Cox model, visualized as a nomogram. The model integrates preoperative PET parameters with postoperative pathology, serving as a posttransplant risk stratification tool rather than a purely preoperative aid, guiding postoperative surveillance intensity and adjuvant therapy planning after pathological microvascular invasion (MVI) confirmation. Model performance was assessed using area under the curve (AUC), calibration curves, and decision curve analysis.

**Results:**

Postoperative recurrence occurred in 80 of 176 patients (45.5%). Univariate analysis revealed that various clinicopathological factors, including PIVKA-II > 40 mAU/mL, alpha-fetoprotein (AFP) > 100 ng/mL, and tumor diameter ≥ 5 cm, as well as PET/CT metabolic parameters such as maximum standardized uptake value (SUVmax), metabolic tumor volume (MTV), and TLG, were significantly associated with recurrence (all *P* < 0.05). Some PET parameters exhibited high collinearity (VIF > 10), and the Boruta algorithm selected five core clinicopathological variables. In multivariate analysis, positive MVI, elevated COV, and elevated TLG remained independent risk factors (all *P* < 0.05), and the model’s concordance index (C-index) was 0.707. The nomogram could predict 1-, 2-, 4-, and 5-year recurrence-free survival (RFS) probabilities. Internal validation demonstrated AUCs of 80.0%, 83.5%, and 81.7% for predicting recurrence at 24, 48, and 60 months, respectively. At 48 months, the calibration curve closely matched the ideal diagonal (slope = 0.96, 95% CI: 0.89-1.03), and decision curve analysis confirmed significant net clinical benefit across threshold probabilities of 10% to 60%.

**Conclusion:**

A simplified model using preoperative PET/CT metabolic parameters (COV, TLG) and MVI predicts HCC recurrence after liver transplantation with good discrimination, calibration, and clinical utility. It assists in precise risk assessment and individualized follow-up and is designed for postoperative surveillance rather than pre-transplant selection.

## Introduction

Hepatocellular carcinoma (HCC) is a highly prevalent primary liver malignancy worldwide and is one of the leading causes of cancer-related mortality. Liver transplantation is a potentially curative treatment option for eligible patients; however, its long-term success is substantially limited by postoperative tumor recurrence (1). The marked heterogeneity in recurrence risk poses considerable challenges to clinical decision-making. Therefore, precise identification of patients at high risk of recurrence after transplantation is crucial for optimizing postoperative surveillance strategies and clarifying the need for adjuvant therapy.

Currently, recurrence risk assessment after liver transplantation for HCC in clinical practice relies primarily on morphological imaging criteria, such as the Milan criteria, and selected clinicopathological features (2, 3). However, these criteria have limited predictive performance and fail to fully capture the intrinsic biological heterogeneity of the tumor, which includes proliferative activity, invasive potential, and metabolic characteristics—core determinants of recurrence risk. As a functional imaging modality, fluorodeoxyglucose (^18^F-FDG) positron emission tomography/computed tomography (PET/CT) can non-invasively assess tumor glucose metabolism, and its baseline metabolic parameters have been shown to be independent predictors of survival after liver transplantation (4, 5).

Integrating multi-dimensional information to construct a predictive model is essential for individualized risk stratification. The nomogram, as an intuitive graphical tool, translates multivariate regression results into a clinically user-friendly scoring system and has been widely and effectively applied in the field of tumor prognosis prediction (6). In gastric cancer studies, the nomogram provides a quantitative basis for decision-making on postoperative adjuvant chemotherapy (7), and in lymphoma, the nomogram can effectively predict the risk of cancer-specific death in patients (8). These models have been shown to outperform traditional staging systems across various solid tumors. In the specific context of liver transplantation for HCC, existing studies have mostly focused on the prognostic value of individual clinicopathological factors or PET metabolic parameters (9), and none have systematically integrated postoperatively available pathological features and PET metabolic heterogeneity parameters to construct a comprehensive nomogram model through rigorous statistical methods.

In summary, although ^18^F-FDG PET/CT metabolic parameters have demonstrated predictive potential, single-modality power is limited. This study aimed to systematically integrate clinicopathological features and PET metabolic parameters (including heterogeneity indices) to construct and validate a nomogram for predicting recurrence after liver transplantation. The model is intended for postoperative risk stratification to guide surveillance and adjuvant therapy decisions, rather than for pretransplant selection, because it relies on MVI status available only from pathology.

## Methods

### Research subjects

This study was a single-center, retrospective cohort study that strictly adhered to the ethical principles of the Declaration of Helsinki and its subsequent revisions. Data were derived from the electronic medical record system, imaging archiving system, and pathological database of patients with HCC who underwent allogeneic liver transplantation at the Liver Transplantation Center of Shulan Hospital from January 2019 to May 2024. The study protocol was approved by the Institutional Ethics Review Board of Shulan Hospital and the requirement for patient informed consent was waived. The inclusion criteria were as follows (1): postoperative pathological confirmation of HCC (2); completion of a preoperative ^18^F-FDG PET/CT examination at this center within 6 weeks before surgery (3); receipt of liver transplantation as the first radical treatment (4); availability of complete preoperative clinical data, postoperative pathological reports, and regular follow-up records. Exclusion criteria included (1): presence of other primary malignancies (2); prior receipt of any preoperative local or systemic anti-tumor treatment for HCC (e.g., TACE, ablation, targeted therapy) (3); poor PET/CT image quality or loss of original data precluding accurate measurement of metabolic parameters (4); follow-up duration of less than 6 months without occurrence of recurrence.

### Indications for PET/CT examination

At this center, preoperative PET/CT is not a routine test for HCC liver transplant candidates. Its use is based primarily on the judgment of clinicians, and common indications include: evaluation of suspected extrahepatic metastases, tumors with unclear biological behavior (e.g., abnormally elevated alpha-fetoprotein (AFP) without definite lesion on conventional imaging), or as a component of a more comprehensive staging assessment for patients beyond the Milan criteria. This selective use may introduce selection bias, as the study population likely represents a higher-risk cohort enriched for aggressive tumor biology; this should be considered when interpreting and generalizing the findings.

### Data collection

Research data were extracted from electronic medical records, imaging archiving systems, and pathological databases, covering five core categories of information (1): Preoperative baseline characteristics, including age, sex, and hepatitis B virus infection status (defined as positive HBsAg) (2); Preoperative clinical and laboratory characteristics, including Child-Pugh classification of liver function, serum AFP level, and des-γ-carboxyprothrombin (PIVKA-II) level. AFP was analyzed as both a continuous and a dichotomous variable (≤100 ng/mL vs. > 100 ng/mL) (3); Postoperative pathological features and transplantation criteria, encompassing tumor morphology (maximum tumor diameter, number of tumors), tumor biology (Edmondson-Steiner differentiation grade, microvascular invasion [MVI] status), tumor T stage, and Milan criteria.

### PET/CT acquisition and reconstruction

All ^18^F-FDG PET/CT examinations were performed using a Siemens Biograph mCT scanner. Patients were instructed to fast for at least 6 hours before the examination, and blood glucose levels were measured before injection; only patients with a blood glucose level < 11.1 mmol/L (200 mg/dL) were included to ensure consistent glucose metabolism conditions. The uptake time ranged from 55 to 65 minutes after ^18^F-FDG injection (3.7–5.5 MBq/kg), with a median of 60 minutes. PET acquisition covered from the vertex to the mid-thigh, with 2–3 minutes per bed position depending on patient weight. Images were reconstructed using an ordered subset expectation maximization (OSEM) algorithm (3 iterations, 21 subsets, matrix size 200 × 200, slice thickness 3 mm, and a Gaussian filter with full-width at half-maximum [FWHM] of 5 mm). Attenuation correction was performed using low-dose CT (120 kVp, automatic tube current modulation [CareDose 4D], with a quality reference mAs: 40 mAs). Scatter and random corrections were applied using standard vendor-supplied algorithms. SUV values were normalized to body weight. The acquisition and reconstruction protocols were in accordance with the European Association of Nuclear Medicine (EANM) and Society of Nuclear Medicine and Molecular Imaging (SNMMI) guidelines for quantitative PET imaging.

### PET/CT metabolic parameter measurement

To quantify metabolic parameters from ^18^F-FDG PET/CT, blinded measurements were performed by two nuclear medicine physicians with more than 5 years of experience, who independently analyzed images using a dedicated workstation (Syngo.via, Siemens Healthineers) while blinded to patient clinical outcomes; any disagreements were resolved by consensus. During the analysis, the relevant metabolic parameters were extracted by manually delineating three-dimensional volumes of interest (3D-VOI) for each intrahepatic HCC lesion on the PET images, using co-registration to CT images for anatomical guidance. For patients with multiple lesions, the lesion with the highest maximum standardized uptake value (SUVmax) was selected for analysis, as it was considered to represent the most biologically aggressive component. The extracted basic parameters included the mean standardized uptake value (SUVmean), its standard deviation (SUVsd), and the maximum SUV (SUVmax). Subsequent derivative parameters were calculated based on these values. Metabolic tumor volume (MTV) was defined using a 40% SUVmax threshold (MTV40), and tumor boundaries were manually adjusted when FDG uptake was close to liver background to ensure accurate delineation. Necrotic areas, bile ducts, vessels, and adjacent bowel activity were carefully excluded. Lesion boundaries were defined on fused PET/CT images, with CT images providing anatomical localization and helping to exclude physiological uptake from adjacent structures. The specific parameters and formulas were as follows: Total lesion glycolysis (TLG) = MTV_40_× SUVmean, where MTV is reported in cm³ and SUVmean is the mean SUV within the threshold-defined MTV region. Coefficient of variation (COV) = SUVsd/SUVmean. Tumor-to-liver ratio (TLR) = tumor SUVmax/liver SUVmax, where liver SUVmax was measured from a spherical ROI (3 cm in diameter) placed in normal-appearing liver parenchyma, avoiding vessels, bile ducts, and necrotic regions; Tumor-to-mediastinal blood-pool ratio (TMR) = tumor SUVmax/mediastinal blood-pool SUVmax, where mediastinal blood-pool SUVmax was measured from a spherical ROI (2 cm in diameter) placed in the descending aorta at the aortic arch level, avoiding the vessel wall and adjacent structures (10). The heterogeneity index (HI) was calculated based on the absolute value of the linear regression slope of MTV at SUVmax thresholds of 40%, 60%, and 80%, as described previously (11).

For the liver background ROI, measurements were performed in normal-appearing liver parenchyma within segments without visible tumor involvement, avoiding vessels, bile ducts, and areas of abnormal uptake. In patients with cirrhosis, regions with heterogeneous uptake were noted, and efforts were made to select relatively homogeneous regions for background measurement. The reproducibility of background ROI placement was assessed as part of the interobserver agreement analysis.

In addition, pre-graft bridging treatment and outcome data were also extracted. The primary outcome was recurrence-free survival (RFS), defined as the time from transplant surgery to tumor recurrence; patients without recurrence were censored at the last follow-up date.

### Sample size estimation

To ensure model stability and avoid overfitting, the present study followed the general criteria for sample size in predictive model studies. For the Cox proportional hazards model, based on the event count (EPV) criterion, the requirement is that the number of outcome events (i.e., the number of relapsed patients) should be at least ten times the number of independent variables that eventually entered the multivariable model (EPV ≥ 10). This study aimed to construct a predictive model that integrates clinicopathological and imaging features, and approximately 7–8 predictors were expected to enter the final model; therefore, the minimum number of recurrence events required is approximately 70-80. Given that patients who undergo ^18^F-FDG PET/CT examination are often selected because of high-risk characteristics and may have a higher risk of postoperative recurrence than the general liver transplant population, the recurrence rate in this cohort was estimated at approximately 40%-50%. To obtain at least 80 recurrence events, the required total sample size was calculated as approximately 80/0.45 ≈ 178 cases. Considering that a small number of cases might have incomplete data in the retrospective study, the initial plan was to include ≥190 consecutive patients to meet the sample size requirements for model development and internal validation.

Ultimately, a total of 176 patients were included in the analysis in this study, of whom 80 had recurrent events. Eight independent predictors were finally included in the multivariate Cox model during the model construction phase. Therefore, the ratio of events to variables in this study is 80/8 = 10, meeting the statistical requirement of EPV ≥ 10, which indicates that the sample size was sufficient to support the robust development and internal validation of a preliminary prediction model.

### Statistics and analysis

All statistical analyses were performed using R software (version 4.3.0). A two-sided *P* value < 0.05 was considered statistically significant. For data presentation, continuous variables with a normal distribution are expressed as mean ± standard deviation, those with a non-normal distribution as median (interquartile range [IQR]), and categorical variables are expressed as frequency (percentage). Variables with missing rates exceeding 20% were excluded from the primary analysis. All cohort patients were divided into two groups according to recurrence status for baseline characteristics description. Independent sample *t*-tests or Mann-Whitney U tests were used for comparisons of continuous variables between groups, and chi-square tests or Fisher’s exact tests for categorical variables. Univariate Cox proportional hazards regression analysis was applied to all clinicopathological features and PET/CT metabolic parameters to identify those significantly associated with the risk of postoperative recurrence.

To address multicollinearity, a collinearity diagnostic test was first performed on all 23 candidate variables (including clinicopathological indicators and PET/CT parameters); a variance inflation factor (VIF) > 10 was defined as severe multicollinearity, and such variables were forcibly excluded. After VIF elimination, univariate Cox regression was rerun on the remaining variables (VIF < 10), and candidate predictors were further screened using a threshold of *P* < 0.1.

On this basis, a differentiated feature selection strategy was adopted (1): Because of the large number of clinicopathological indicators, the Boruta algorithm was used to objectively evaluate the correlation of each indicator with recurrence, and core predictors were selected (2); For PET/CT metabolic parameters, after VIF elimination and univariate P-value screening, representative indicators with low multicollinearity that captured two distinct dimensions—metabolic burden (TLG) and metabolic heterogeneity (COV)—were chosen for the final model, while other collinear parameters were excluded.

Model performance evaluation and internal validation involved multi-dimensional comprehensive analysis: The area under the curve (AUC) at 24, 48, 60 months was calculated using the time-dependent receiver operating characteristic (ROC) curve, and the concordance index (C-index) was verified via 1000 Bootstrap resampling to assess discrimination. A bootstrap-corrected calibration curve was plotted to evaluate agreement between predicted probabilities and actual observations, and the calibration slope was computed as a quantitative metric. Clinical utility was quantified by decision curve analysis (DCA), the threshold range for guiding clinical decisions was defined. To further evaluate model robustness, k-fold cross-validation (5-fold and 10-fold, each with 100 iterations) was performed. To quantify the incremental value of PET parameters, nested models were compared using the concordance index (C-index), integrated discrimination improvement (IDI), and net reclassification improvement (NRI).

## Results

### Clinicopathological characteristics of the patients

A total of 176 liver transplant patients with HCC who underwent preoperative ^18^F-FDG PET/CT examination were included. The median follow-up duration was 12 months (range: 6–61 months). Recurrence occurred in 80 patients (45.5%). Comparisons of clinicopathological features (**Table 1**) showed that patients with recurrence had more aggressive tumor biological characteristics. Regarding serum tumor markers, the proportion of patients with PIVKA-II > 40 mAU/mL was significantly higher in the recurrence group than that in the non-recurrence group (90.8% vs. 72.9%, *P* = 0.003), and the proportion with AFP > 100 ng/mL was also higher (48.8% vs. 30.2%, *P* = 0.012). In terms of postoperative pathological features, the proportions of patients with tumor maximum diameter ≥ 5 cm (57.5% vs. 33.3%, *P* = 0.001), positive MVI (75.0% vs. 41.7%, *P* < 0.001), and T3-T4 stage (52.5% vs. 32.3%) and those exceeding the Milan criteria (83.3% vs. 59.4%, *P* < 0.001) were all significantly higher. However, there were no statistically significant differences between the two groups in terms of age, gender, Child-Pugh classification of liver function, hepatitis B virus infection rate, tumor multiplicity, differentiation grade, or proportion receiving bridging treatment.

**Table 1:** Baseline clinicopathological characteristics of the patients.

Characteristics	No recurrence (N=96)	Relapse (N=80)	P value
Demographic characteristics			
Age (years), mean ±SD	54 ± 10	53 ± 10	0.438
Male, n (%)	89 (92.7)	72 (90.0)	0.522
Serum tumor markers			
PIVKA-II > 40 mAU/mL, n (%)	70 (72.9)	69 (90.8)	0.003
AFP > 100 ng/mL, n (%)	29 (30.2)	39 (48.8)	0.012
Liver function			
Child-Pugh classification, n (%)			0.665
A	78 (81.3%)	67 (83.8%)	
B	18 (18.8%)	13 (16.3%)	
Hepatitis B virus infection, n (%)	94 (97.9)	80 (100.0)	0.501
Tumor pathological features			
Tumor maximum diameter ≥ 5 cm, n (%)	32 (33.3)	46 (57.5)	0.001
Multiple tumors, n (%)	41 (42.7)	41 (51.3)	0.258
MVI positive, n (%)	40 (41.7)	60 (75.0)	<0.001
Degree of differentiation (high), n (%)	6 (6.3)	3 (3.8)	0.513
Pathological T stage (T3-T4), n (%)	31 (32.3)	42 (52.5)	0.007
Beyond the Milan criteria, n (%)	57 (59.4)	65 (83.3)	<0.001
Treatment-related			
Receive pregraft bridging therapy, n (%)	58 (60.4)	54 (67.5)	0.331

Continuous variables were compared between groups using independent sample t-tests or Mann-Whitney U tests; Categorical variables were analyzed using chi-square tests or Fisher's exact tests. Abbreviation: MVI (Microvascular invasion).

### PET/CT metabolic parameter assessment of the patients

Intergroup comparisons of preoperative PET/CT metabolic parameters are shown in **Table 2**. For metabolic intensity, the median SUVmax, SUVmean and SUVpeak were significantly higher in the recurrence group than in the non-recurrence group (all *P* < 0.01). For metabolic volume and burden, MTV and TLG were also significantly greater in the recurrence group (all *P* < 0.05). In addition, the HI, which reflects the heterogeneity of intratumoral metabolism, was higher in the recurrence group (*P* = 0.030). Two standardized metabolic ratios—TLR and TMR—were also significantly elevated in the recurrence group (both *P* < 0.001). However, no significant difference was found in the COV of metabolism within the tumor between the two groups.

**Table 2:** Comparison of preoperative ^18^F-FDG PET/CT metabolic parameters.

Parameters	No recurrence (N=96)	Relapse (N=80)	"P value
Metabolic intensity			
SUVmax	4.18 (3.38, 5.95)	5.44 (4.22, 7.56)	<0.001
SUVmean	2.58 (2.24, 3.26)	3.07 (2.64, 4.25)	0.004
SUVpeak	3.47 (2.84, 4.82)	4.46 (3.61, 6.43)	<0.001
SUVsd	0.51 (0.33, 0.76)	0.71 (0.43, 1.04)	0.003
Metabolic volume and load			
MTV_40_	47 (19, 140	91 (27, 271)	0.039
TLG	124 (52, 373)	300 (69, 856)	0.022
Heterogeneity parameters			
COV	0.18 (0.14, 0.24)	0.21 (0.14, 0.26)	0.064
HI	1.1 (0.4, 3.4)	2.2 (0.6, 6.6)	0.030
Standardized ratio			
TLR	1.42 (1.16, 1.80)	1.80 (1.41, 2.65)	<0.001
TMR	1.73 (1.42, 2.46)	2.28 (1.73, 3.63)	<0.001

^18^F-FDG (^18^ f-fluorodeoxyglucose), PET/CT (positron emission computed tomography), SUVmax (maximum standard uptake), SUVmean (mean standard uptake), SUVpeak (peak standard uptake), SUVsd (standard deviation), MTV (metabolic tumor volume), TLG Tumor metabolic load, COV (coefficient of variation), HI (heterogeneity index), TLR (tumor-liver ratio), TMR (tumor-mediastinal pool ratio). Continuous variables were compared between groups using the independent sample t-test or the Mann-Whitney U test; Categorical variables were analyzed using chi-square tests or Fisher's exact tests.

### Screening of predictors and collinearity treatment

To avoid information redundancy caused by multicollinearity, collinearity diagnostics were performed on all 23 candidate variables (**Supplementary Table 1**). The results showed that the VIF values for hepatitis B virus infection status, SUVmax, SUVpeak, MTV40, HI, TMR, and TLR were all > 10, indicating severe multicollinearity; these variables were therefore forcibly excluded.

Among the remaining variables with VIF < 10, univariate Cox proportional hazards regression was further performed (**Supplementary Table 2**, derived from **Supplementary Table 1** after removing variables with VIF > 10), and 10 candidate variables were selected using a threshold of *P* < 0.1. These included six traditional clinical factors—PIVKA-II > 40 mAU/mL, AFP > 100 ng/mL, tumor maximum diameter ≥ 5 cm, positive MVI, T stage (T3–T4), and exceeding the Milan criteria—and four PET/CT metabolic parameters: SUVmean, SUVsd, TLG, and COV (COV had *P* = 0.075 and was retained as per the preset *P* < 0.1 criterion).

Further manual selection was guided by the Boruta algorithm results and the clinical meaning of each indicator. Among the traditional clinical factors, the Boruta importance ranking of T stage was lower than that of tumor size and Milan criteria, and all three reflected tumor burden and invasion extent with substantial overlap in clinical information. To avoid multivariate collinearity and overfitting, T stage was excluded. For PET/CT metabolic parameters, SUVmean and SUVsd were related to the same metabolic dimensions as TLG and COV, respectively. Given that TLG integrates metabolic volume and intensity (TLG = MTV × SUVmean), it more comprehensively reflects overall metabolic burden than SUVmean alone; COV, being a mean-normalized heterogeneity index (COV = SUVsd/SUVmean), offers better cross-individual comparability and clinical generalizability than the absolute dispersion value SUVsd. Therefore, TLG and COV were retained, while SUVmean and SUVsd were excluded.

### Multivariable model construction: independent contributions of clinicopathology and PET parameters

Based on the above-described differentiated screening strategies, a multivariable Cox proportional hazards regression model was constructed. Ultimately, eight variables were included: age (mandatorily), MVI, Milan criteria, tumor maximum diameter ≥ 5 cm, PIVKA-II > 40 mAU/mL, AFP > 100 ng/mL, COV, and TLG. The event-to-variable ratio was 80:8 = 10:1, meeting the requirements for model robustness. The results of the multivariable analysis are presented in **Table 3**.

**Table 3:** Multivariate analysis of influencing factors (Cox regression).

Characteristic	HR	95% CI	P value
Age, years	1.01	0.98, 1.03	0.584
MVI			
Negative	—	—	—
Positive	2.42	1.36, 4.33	0.003
Milan standard			
Not exceeded	—	—	—
Beyond	1.41	0.66, 3.02	0.374
Tumor size (cm)			
<5	—	—	—
≥5	1.24	0.71, 2.16	0.455
PIVKA-II > 40 mAU/mL			
no	—	—	—
is	1.90	0.85, 4.24	0.119
AFP >100 ng/mL			
no	—	—	—
is	1.28	0.77, 2.12	0.335
COV	9.12	1.37, 60.70	0.022
TLG	10.00	1.00, 100.00	0.039

Model fitting key metrics: C-index=0.707; r-squared = 0.985; Log-likelihood = -336; AIC = 689; BIC = 707.

After adjusting for the effects of other variables, positive MVI (*P* = 0.003), higher COV (*P* = 0.022), and higher TLG (*P* = 0.039) remained independent risk factors for recurrence after liver transplantation. Notably, the hazard ratio for COV was 9.12 (95% CI: 1.35–61.74); although the wide confidence interval indicates some uncertainty in this estimate. Other clinicopathological features screened out by the Boruta algorithm (Milan criteria, tumor size, PIVKA-II, AFP) did not reach independent statistical significance in this multivariable model. The C-index of the model was 0.707.

To evaluate the incremental value of PET parameters, three nested Cox models were compared: a clinical-only model (MVI, Milan criteria, tumor size ≥ 5 cm, PIVKA-II > 40, AFP > 100 ng/mL), a PET-only model (COV, TLG, age), and the combined model. The combined model achieved a C-index of 0.707 (95% CI: 0.668–0.746), compared with 0.652 (95% CI: 0.609–0.695) for the clinical-only model and 0.634 (95% CI: 0.590–0.678) for the PET-only model. The IDI for the combined model versus the clinical-only model was 0.087 (95% CI: 0.041–0.133, *P* < 0.001), and the NRI was 0.214 (95% CI: 0.098–0.330, *P* < 0.001), indicating a significant improvement in risk classification.

To further evaluate model robustness and mitigate overfitting concerns, k-fold cross-validation was performed. In 5-fold cross-validation (100 iterations), the mean C-index was 0.695 (95% CI: 0.652–0.738), and in 10-fold cross-validation, it was 0.699 (95% CI: 0.657–0.741). The calibration slopes across folds ranged from 0.92 to 1.08, indicating good model stability. These findings suggest that the model was not substantially overfitted despite the relatively small sample size.

### Multi-factor model construction nomogram and model evaluation

**Figure 1:**
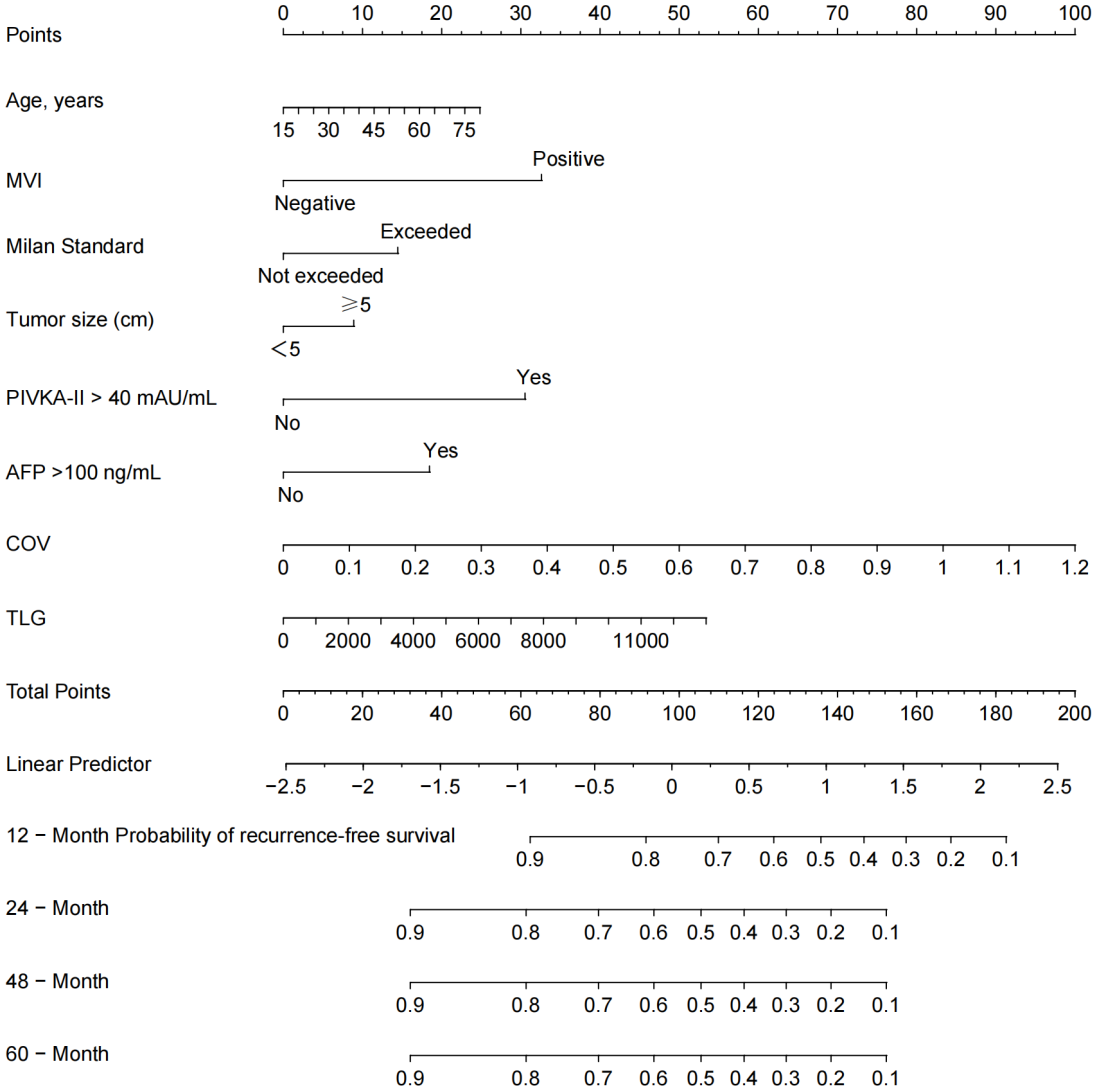
Nomogram for predicting RFS after liver transplantation for HCC. This nomogram was used for individualized prediction of RFS probabilities for patients at 1 year, 2 years, 4 years, and 5 years after surgery. It is used as follows: Determine the variable score: On the “Points” scale above, determine the corresponding score based on the values of each indicator of the patient. Calculate the Total score: Add up the scores of all variables to get “Total Points”. Prediction probability: Project “Total Points” onto the “Linear Predictor” ruler at the bottom or directly to the “RFS prediction probability” at the bottom to read out the corresponding prediction probability.

Based on the final multivariate Cox regression model, a nomogram was constructed for predicting individualized RFS probability (**Figure 1**).

The integrated model included MVI, Milan criteria compliance, maximum tumor diameter, PIVKA-II > 40 mAU/mL, AFP > 100 ng/mL, and the PET metabolic parameters COV and TLG, and provided predictions of RFS at 1 year, 2 years, 4 years, and 5 years after surgery. A clinical example is provided in the **Supplementary Materials** to demonstrate nomogram use: For a patient with COV = 0.15, TLG = 150 cm³, positive MVI, and other variables at reference levels, the total points are calculated, and the corresponding 4-year RFS probability is approximately 62%. Specifically, consider a patient with COV = 0.15, TLG = 150 cm³, positive MVI, tumor diameter < 5 cm, within Milan criteria, PIVKA-II ≤ 40 mAU/mL, and AFP ≤100 ng/mL. Using the nomogram, the total points would be calculated as follows: MVI (+) = 65 points, COV = 0.15 ≈ 40 points, TLG = 150 ≈ 35 points, yielding a total of approximately 140 points. This corresponds to a 4-year RFS probability of approximately 62%.

**Figure 2:**
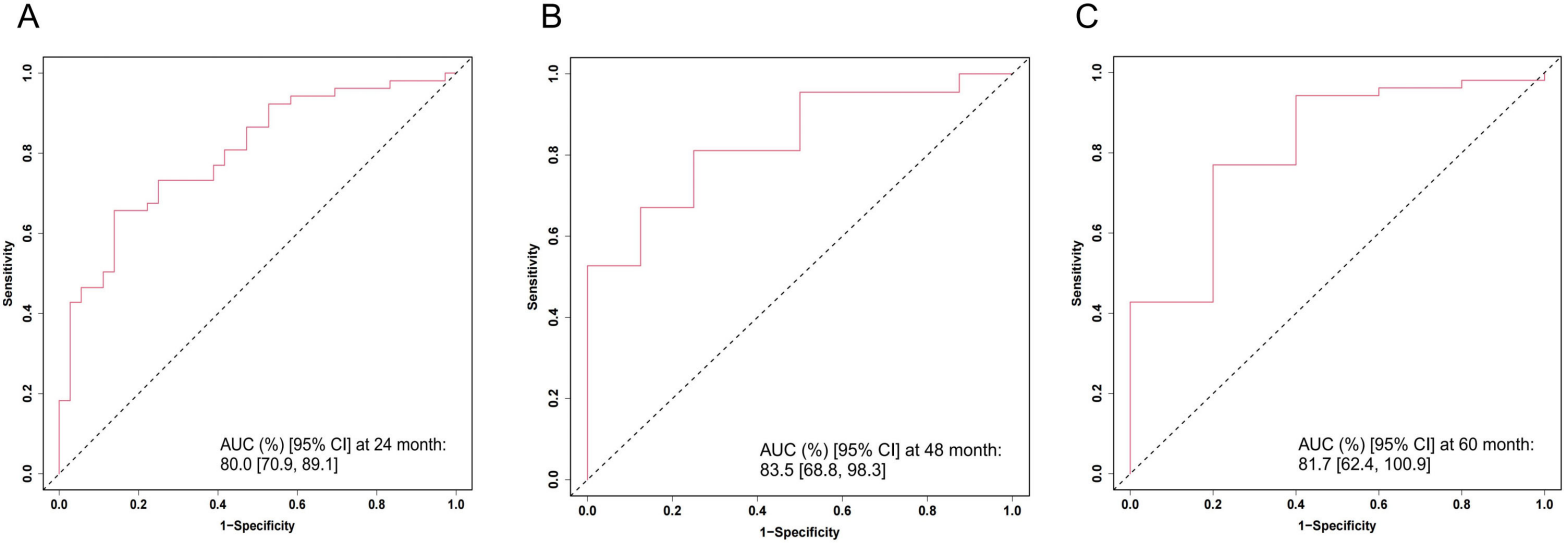
Time-dependent ROC curve of the nomogram model for predicting recurrence risk. **(A)** ROC curve for predicting recurrence at 24 months after surgery. **(B)** ROC curve for predicting recurrence at 48 months after surgery. **(C)** ROC curve for predicting recurrence at 60 months after surgery.

To evaluate model predictive performance, internal validation was conducted. The time-dependent ROC curve (**Figure 2**) showed that the AUCs for predicting recurrence at 24, 48, and 60 months were 80.0% (95% CI: 70.9–89.1), 83.5% (95% CI: 68.8–98.3), and 81.7% (95% CI: 62.4–100.9), respectively, indicating good discriminatory ability during the 48-month follow-up period.

However, the wide 95% confidence interval at 60 months reflects the limited number of patients still at risk at this late time point because of the median follow-up of 12 months; therefore, long-term predictions beyond 48 months should be interpreted with caution. The model is best suited for predicting recurrence risk within 24 to 48 months after transplantation. The calibration curve at 48 months showed excellent agreement between predicted and observed RFS, with a calibration slope of 0.96 (95% CI: 0.89–1.03) and an intercept of −0.02 (95% CI: −0.11 to 0.07). A slope close to 1 indicates that the predicted risks are well-calibrated without systematic overestimation or underestimation.

**Figure 3:**
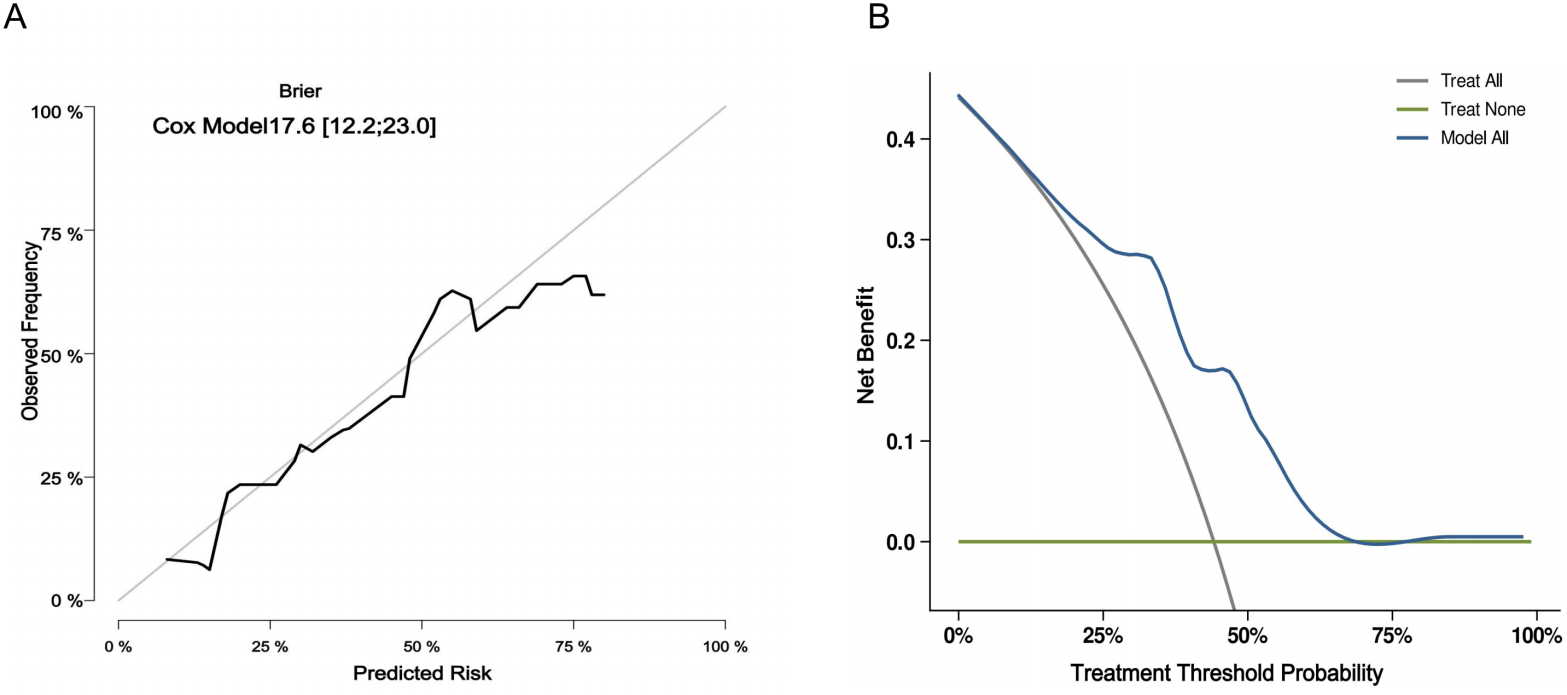
Calibration curve versus decision curve analysis of the nomogram model at 48 months after surgery. **(A)** Calibration curve. The graph shows the calibration of the nomogram model for predicting RFS at 48 months after surgery. The diagonals (gray dotted lines) in the figure represent ideal perfect calibration. The blue solid line represents the calibration curve of this model, which fits the diagonal closely. After internal validation and correction using the Bootstrap method (1000 resampling), the calibration curve (blue dotted line) remained very close to the diagonal, indicating good consistency between the predicted probabilities of the model and the actual observed 48-month recurrent-free survival rate. **(B)** Decision curve analysis. The graph evaluated the net benefit of using this nomogram model to assist in clinical decision-making at a time point of 48 months after surgery. Three strategies are shown in the figure: “All intervention” (gray dotted line), “all no intervention” (black dotted line), and “Intervention based on the nomogram model” (blue solid line).

Further analysis confirmed the predictive accuracy of the model (**Figure 3A**): at 48 months after surgery, the calibration curve closely adhered to the ideal diagonal, indicating a high degree of consistency between the predicted probabilities and the actual observations. Decision curve analysis (**Figure 3B**) confirmed its clinical utility: when the threshold probability for clinical decisions was set within a reasonable range of 10% to 60%, using the model to guide clinical decisions (e.g., enhanced surveillance) provided significant net benefit.

## Discussion

This study successfully constructed and validated a nomogram model for predicting the risk of recurrence after liver transplantation for HCC by integrating ^18^F-FDG PET/CT metabolic parameters (acquired preoperatively) with postoperatively available clinicopathological features. The results showed that postoperatively confirmed positive MVI, elevated metabolic COV, and elevated TLG were independent risk factors for tumor recurrence, and the model based on these factors demonstrated good discrimination, calibration, and clinical utility. This finding underscores the value of integrating functional imaging parameters that reflect tumor metabolic activity and heterogeneity with traditional clinicopathological assessment for more accurate prognosis estimation of liver transplant recipients with HCC. The model is intended for use after liver transplantation to guide postoperative surveillance intensity, rather than for preoperative recipient selection, because it relies on MVI status which is available only from postoperative pathology.

The present study adopted a strategy of performing global VIF elimination first, followed by univariate screening, which was more cautious than the conventional approach of screening first and then diagnosing collinearity—collinearity may distort the univariate effect estimates of some variables. Performing VIF diagnosis first helps avoid erroneously retaining collinear variables in subsequent analysis. On this basis, although T stage had a significant univariate *P* value, its importance ranking in the Boruta algorithm was relatively low among all traditional clinical indicators, and it was excluded because its clinical connotation overlaps with that of tumor size and Milan criteria, both of which reflect tumor burden. Among PET parameters, TLG and COV represent overall metabolic burden and standardized intratumoral heterogeneity, respectively, and they are more informative and offer better cross-individual comparability than SUVmean alone or absolute dispersion values such as SUVsd. This strategy enabled the final model to cover five dimensions – pathological invasion, clinical stage, serology, metabolic burden, and metabolic heterogeneity – with fewer variables, while balancing statistical robustness and clinical operability.

Tumor recurrence remains a major obstacle to long-term survival after liver transplantation, and its root cause lies in the pre-existing micrometastases or more aggressive tumor biological characteristics (12). In the present cohort, patients in the recurrence group had a higher proportion of MVI, exceeding the Milan criteria (13), and higher levels of serum AFP and PIVKA-II (14), findings that are consistent with previous studies and point to a more aggressive tumor phenotype. Notably, while most of the conventional PET metabolic parameters were associated with recurrence in the univariate analysis, only TLG and COV retained independent predictive value after controlling for confounding factors. TLG, as a composite indicator integrating metabolic intensity and volume, may more comprehensively characterize the overall metabolic load of the tumor (15), while COV, a measure of heterogeneity (16), showed no significant difference in univariate analysis (median [IQR] 0.14 [0.10-0.18] in the recurrence group vs. 0.12 [0.09–0.16] in the non-recurrence group, *P* = 0.064) but exhibited a hazard ratio as high as 9.12 in the multivariable model.

An apparent statistical paradox was observed: COV was not significantly different between recurrence groups in univariate analysis (*P* = 0.064) but became an independent risk factor in the multivariate model (HR = 9.12, *P* = 0.022). This discrepancy is likely attributable to a suppressor effect and interactions with other covariates, particularly TLG and tumor size. Furthermore, COV may interact with MVI status, with the heterogeneity signal being more pronounced in MVI-positive tumors. The wide confidence interval of the HR (1.35–61.74) should be noted, as it indicates limited precision of this estimate and warrants cautious interpretation. This finding suggests that metabolic heterogeneity represents a deeper prognostic indicator whose predictive value may be masked by overall metabolic activity in unadjusted analyses. The reproducibility of COV (ICC = 0.82) was slightly lower than that of other parameters, which may contribute to the wider confidence interval and should be considered when interpreting its clinical utility. Standardization of VOI delineation protocols and automated segmentation methods could improve reproducibility for clinical implementation.

Compared with previous studies, this study has expanded both methodological and predictive dimensions. Numerous studies have focused on using clinical features, serum markers, or conventional imaging features to predict the risk of recurrence of HCC (17, 18). In recent years, radiomics based on CT or MRI has shown potential in predicting MVI and prognosis (19, 20). For example, some studies have achieved high diagnostic efficacy in predicting MVI by using peritumoral radiomics features of gadoxetic acid disodium-enhanced MRI (21). Another study that combined MRI habitat imaging with clinical features also produced a model that performed well in predicting MVI and early recurrence (20). However, these methods relied mainly on morphology and enhancement patterns. ^18^F-FDG PET/CT can reveal the biological behavior of tumors at the functional level by providing information on their glucose metabolism. Previous studies have confirmed that parameters such as SUVmax are associated with the prognosis of HCC (22, 23). This study not only confirmed the value of metabolic intensity indicators but also deepened the understanding of metabolic characteristics by including comprehensive metabolic parameters (e.g., TLG) and heterogeneity parameters (e.g., COV). Particularly noteworthy is the independent predictive effect of COV, which partly echoes the findings of a recent study specifically targeting the prediction of MVI before liver transplantation, which also found that the heterogeneity index is one of the independent predictors of MVI (24), however, this study did not directly link it to long-term recurrence. The present study directly linked COV to the recurrence outcome after liver transplantation and revealed its strong predictive power in a multivariable model, which may represent an important addition to the clinical significance of tumor heterogeneity. Heterogeneity may reflect clonal diversity within the tumor, hypoxic regions or necrosis, all of which are associated with enhanced treatment resistance and metastatic potential (25, 26).

It is important to acknowledge the inherent limitations of FDG PET/CT in HCC. FDG uptake in HCC is heterogeneous; well-differentiated HCC lesions may show low or absent FDG uptake, whereas poorly differentiated, more aggressive tumors or those with MVI are more likely to be FDG-avid. This dual feature means that FDG PET/CT may help identify aggressive tumor biology but has limited sensitivity for low-FDG-avid lesions. In the present cohort, a proportion of HCC lesions showed low FDG uptake, which may have led to an underestimation of recurrence risk in some patients. FDG PET/CT is therefore better positioned as a high-risk biological screening tool rather than a pure lesion-detection tool for HCC. Its advantage over MRI, contrast-enhanced CT, or Gd-EOB-DTPA MRI lies in metabolic phenotyping rather than morphologic resolution. Future studies incorporating other PET tracers targeting complementary tumor biology pathways may further improve phenotyping and recurrence prediction. FDG PET/CT reflects tumor glycolytic metabolism and aggressive metabolic phenotype, whereas FAPI PET/CT may reflect stromal activation and fibrosis-related tumor microenvironment; combining the two modalities may improve risk stratification for recurrence after liver transplantation for HCC.

The strengths of this study include the prospective systematic evaluation of multiple PET metabolic parameters and the use of modern feature selection methods such as the Boruta algorithm to handle the high collinearity, thereby extracting core predictors and enhancing model robustness and interpretability. The incremental value analysis (IDI = 0.087, NRI = 0.214) confirmed that adding PET parameters significantly improved risk classification beyond clinical factors alone. The final nomogram integrates key indicators in an intuitive graphical way, facilitating individualized risk assessment by clinicians. Internal validation demonstrated good and stable predictive performance, with AUCs for predicting recurrence at 2 to 5 years after surgery exceeding 80%, and the calibration curve at 48 months showed excellent agreement (calibration slope = 0.96). Cross-validation (5-fold and 10-fold) yielded mean C-indices of 0.695 and 0.699, respectively, further supporting model stability. Decision curve analysis further confirmed that the model could provide net benefit to clinical decision-making over a wide range of threshold probabilities, suggesting that guiding postoperative monitoring or adjuvant treatment strategy adjustments based on the model is expected to benefit the overall patient population.

However, several limitations should be acknowledged. First, this was a single-center retrospective study with a relatively limited sample size and a median follow-up of 12 months, which may not be sufficient for comprehensive assessment of HCC recurrence, especially advanced recurrence. It should be noted that the 60-month AUC had a wide 95% confidence interval (62.4%–100.9%), reflecting the limited number of patients at risk at this late time point due to the median follow-up of 12 months. Therefore, while the model shows good discriminative ability, long-term predictions (beyond 48 months) should be interpreted with caution. The model is best suited for predicting recurrence risk within 24 to 48 months after transplantation. Despite good internal validation performance, external validation in independent cohorts is lacking, and the generalizability of the model in different populations and different medical centers remains to be confirmed. External validation will be needed in prospective cohorts with multiple centers and large samples in the future to establish its wide applicability. Second, although the study included a large number of PET parameters, it did not involve emerging molecular imaging probes such as ^68^Ga-FAPI PET/CT targeting fibroblast activation protein. Studies have shown that ^68^Ga-FAPI PET/CT may have higher sensitivity in the detection of HCC, especially in the context of liver cirrhosis (15). In this context, FDG and FAPI PET/CT provide complementary information: FDG reflects tumor glycolytic metabolism and the aggressive metabolic phenotype, whereas FAPI may reflect stromal activation and the fibrosis-related tumor microenvironment. Combining the two modalities could improve recurrence risk stratification and may offer incremental predictive value beyond FDG-derived metrics. Future research should explore whether such novel probe parameters can be integrated into the current model to further enhance its performance. Furthermore, this study mainly focused on the characteristics of the tumor itself, with relatively insufficient consideration of the host immune microenvironment and the state of systemic inflammation. In addition, no molecular typing or gene expression analysis of recurrent tumors was conducted in this study.

Clinical implications: The clinical significance of this study lies in providing a practical tool for individualized risk assessment after liver transplantation for HCC. Because the model relies on MVI status, which is only available from postoperative pathology, its intended use is to guide postoperative surveillance intensity rather than preoperative recipient selection. The recurrence risk calculated based on the nomogram helps clinicians identify high-risk patients after pathological confirmation. For very high-risk patients, more aggressive preoperative downstaging therapy (such as combined immunotherapy (27)), may be considered before transplantation, while closer postoperative monitoring or exploration of the feasibility of adjuvant therapy where conditions permit may be guided by the model after pathological confirmation. At the same time, the model also aids in patient counseling and adherence management. Future research directions should focus on the following: first, conducting multi-center external validation and attempting to make the model available online for clinical use; second, exploring fusion with MRI functional imaging or emerging PET probes (28) to construct multimodal predictive models; third, attempting to incorporate circulating biomarkers into the predictive system (29) for a more non-invasive assessment; fourth, exploring the linkage between predictive models and treatment decisions, such as guiding individualized adjuvant therapy or immunotherapy based on risk stratification, ultimately achieving precise management of liver transplant recipients with HCC and improving long-term survival outcomes. Additionally, future studies should prospectively evaluate whether integrating preoperative prediction of MVI (using imaging-based models) could allow this postoperative model to be converted into a truly preoperative tool.

## Conclusion

In conclusion, this study confirms that preoperative ^18^F-FDG PET/CT metabolic parameters (particularly COV and TLG) combined with postoperatively available clinicopathological features (MVI) can provide an efficient and streamlined model for predicting the risk of recurrence after HCC liver transplantation. The model is intended for use after liver transplantation to guide postoperative surveillance intensity, rather than for preoperative recipient selection. It helps clinicians develop more targeted postoperative surveillance and intervention plans for high-risk populations, thereby improving the long-term prognosis of patients. Future studies could expand the sample size and conduct multicenter prospective studies to further validate the model’s validity and stability, incorporate more potential predictors, optimize the model structure, and enhance its clinical application value to provide stronger support for the precise diagnosis and treatment of HCC liver transplant patients.

## Data Availability

The original contributions presented in the study are included in the article/**Supplementary Material**. Further inquiries can be directed to the corresponding author.
