# Supplementary material for: A streamlined predictive model for predicting the risk of recurrence after liver transplantation for hepatocellular carcinoma was constructed based on preoperative 18F-FDG PET/CT metabolic parameters and clinicopathological features
Source: Front Oncol. 2026 Jul 27;16:1832413. doi: 10.3389/fonc.2026.1832413 (PMC13453818; doi:10.3389/fonc.2026.1832413)
Supplement: Supplementary file 1 [file DataSheet1.docx]

**Supplement Table 1. Collinearity analysis of the indicators**

| Indicators | VIF | VIF_CI_low | VIF_CI_high |
| --- | --- | --- | --- |
| Age | 1.326084 | 1.153346 | 1.914325 |
| Gender | 1.438566 | 1.772644 | 1.772644 |
| PIVKA-II >40 mAU/mL | 1.51005 | 1.48255 | 1.472544 |
| AFP >100 ng/mL | 1.392082 | 1.24793 | 1.79507 |
| Child-Pugh classification | 1.488229 | 1.285763 | 1.834143 |
| Hepatitis B virus | 568.15252 | 232.560154 | 2658.36645 |
| Tumor size | 4.990665 | 3.962942 | 6.374864 |
| Number of tumors | 1.434381 | 1.245846 | 1.767499 |
| MVI | 1.307632 | 1.153576 | 1.616229 |
| Degree of differentiation | 1.766567 | 1.495071 | 2.186951 |
| T staging | 5.096816 | 4.044369 | 6.513096 |
| Milan Standard | 1.733092 | 1.469735 | 2.144100 |
| Pregraft bridging therapy | 1.324941 | 1.116039 | 1.658967 |
| SUVmax | 24.353287 | 7.870359 | 68.829864 |
| SUVmean | 4.755710 | 1.493359 | 7.914243 |
| SUVpeak | 17.792815 | 2.861705 | 41.316735 |
| SUVsd | 2.023780 | 1.506411 | 5.971493 |
| MTV40 | 200.818356 | 102.163211 | 395.672007 |
| COV | 3.730742 | 5.789026 | 9.474865 |
| TLG | 3.475550 | 4.793390 | 7.428156 |
| HI | 12.835651 | 7.615444 | 54.424645 |
| TMR | 10.849216 | 8.458377 | 13.600055 |
| TLR | 13.914711 | 10.810976 | 18.000324 |
