# Supplementary material for: A streamlined predictive model for predicting the risk of recurrence after liver transplantation for hepatocellular carcinoma was constructed based on preoperative 18F-FDG PET/CT metabolic parameters and clinicopathological features
Source: Front Oncol. 2026 Jul 27;16:1832413. doi: 10.3389/fonc.2026.1832413 (PMC13453818; doi:10.3389/fonc.2026.1832413)
Supplement: Supplementary file 2 [file DataSheet2.docx]

**Supplement Table 2. Results of univariate Cox proportional hazards regression analysis**

| Characteristics | HR | (95%CI) | P value |
| --- | --- | --- | --- |
| Age (continuous variable) | 0.99 | 0.97, 1.02 | 0.685 |
| Gender (male) | 0.90 | 0.36, 2.24 | 0.819 |
| PIVKA-II >40 mAU/mL (yes) | 3.52 | 1.41, 8.79 | 0.007 |
| AFP >100 (yes) | 0.48 | 0.29, 0.78 | 0.003 |
| Hepatitis B virus (yes) | 9,132 | 0.00, Inf | 0.995 |
| Tumor size (≥5 cm) | 2.62 | 1.57, 4.38 | <0.001 |
| Number of tumors (≥2) | 1.29 | 0.79, 2.11 | 0.306 |
| MVI (positive) | 4.06 | 2.20, 7.48 | <0.001 |
| Degree of differentiation (high) | 0.27 | 0.04, 1.92 | 0.189 |
| T staging (T3-T4) | 2.22 | 1.35, 3.64 | 0.002 |
| Milan Standard (beyond) | 5.14 | 2.21, 11.94 | <0.001 |
| Pregraft bridging therapy (yes) | 1.14 | 0.68, 1.90 | 0.627 |
| SUVmean | 1.14 | 1.02, 1.26 | 0.021 |
| SUVsd | 1.41 | 1.03, 1.92 | 0.032 |
| TLG | 1.00 | 1.00, 1.01 | <0.001 |
| COV | 7.83 | 0.81, 75.47 | 0.075 |
| TLR | 1.34 | 1.16, 1.55 | <0.001 |

HR is the risk ratio; 95% confidence intervals. Abbreviation full name: ¹⁸F-FDG (¹⁸ f-fluorodeoxyglucose), PET/CT (positron emission computed tomography), SUVmax (maximum standard uptake), SUVmean (mean standard uptake), SUVpeak (peak standard uptake), SUVsd (standard deviation), MTV (metabolic tumor volume), TLG Tumor metabolic load, COV (coefficient of variation), HI (heterogeneity index), TLR (tumor-liver ratio), TMR (tumor-mediastinal pool ratio).
